# A Lot on Your Plate? Well-to-Well Contamination as an Additional Confounder in Microbiome Sequence Analyses

**DOI:** 10.1128/mSystems.00362-19

**Published:** 2019-06-25

**Authors:** Alan W. Walker

**Affiliations:** a Rowett Institute, University of Aberdeen, Aberdeen, United Kingdom

**Keywords:** contamination, microbiome, sequencing

## Abstract

DNA sequence-based microbiome studies can be impacted by a range of different methodological artefacts. Contamination originating from laboratory kits and reagents can lead to erroneous results, particularly in samples containing a low microbial biomass. Minich and colleagues (mSystems 4:e00186-19, 2019, https://doi.org/10.1128/mSystems.00186-19) report on a different form of contamination, cross-contamination between samples that are processed together. They find that transfer of material between samples in 96-well plates is a common occurrence. The DNA extraction step, particularly when carried out automatedly, is identified as the major source of this contamination type. Well-to-well contamination distorts diversity measures, with low-biomass samples particularly affected. This report has important implications for attempts to decontaminate microbiome sequencing results. As contamination is derived from both external sources and crossover between samples, it is not appropriate to simply remove sequence variants that are detected in negative-control blanks, and more-nuanced decontamination approaches may be required.

## Abstract (precis)

DNA sequence-based microbiome studies can be impacted by a range of different methodological artefacts. Contamination originating from laboratory kits and reagents can lead to erroneous results, particularly in samples containing a low microbial biomass.

## TEXT

The last 15 years have seen a rapid escalation in research on microbial communities, largely driven by the advent and widespread adoption of high-throughput sequencing techniques (1, 2). These sequence-based methodologies have allowed us to characterize the microbial world at scales and depths that would have been unthinkable just a decade or so ago.

While it has been an incredibly productive and exciting time for microbiome research, some of the results generated using sequence-based approaches have been controversial and occasionally contradict conventional knowledge. Studies have “discovered” diverse microbial communities in environments that were previously considered to be largely sterile or have associated unexpected microbes with environments where it is difficult to find plausible explanations for their presence.

It is important, therefore, to emphasize that, while incredibly powerful, DNA sequencing approaches are fundamentally just techniques, and all techniques have biases and limitations (3, 4). Indeed, there are well-described biases introduced during steps such as sample collection and storage, DNA extraction, template amplification, and bioinformatics analyses, all of which have the potential to skew results and subsequent interpretations (3, 5).

A further potential problem with DNA sequence-based microbiome profiling methods is contamination, as the laboratory kits and reagents that are used to process samples for subsequent sequencing are not sterile. Contamination arising from these sources was first reported in the 1990s (6), and more recent work has demonstrated the impact that these contaminants can have on modern microbiome profiling studies (7–12). The impact is particularly dramatic on samples containing a low biomass, as the background level of contamination can effectively “swamp” any underlying real signal in these samples and therefore lead to erroneous conclusions (7).

In addition to external sources of contamination, there can also be cross-contamination between samples within a given study. Certain steps in the process of generating microbiome sequence data can involve numerous samples being processed simultaneously, often in 96-well plates. Cross-contamination is a problem that has been acknowledged by researchers working in the field of microbiome research previously (12), but it remains underreported and largely unquantified.

In a series of experiments, Minich et al. (13) investigated the potential extent of well-to-well contamination in sequence-based microbiome studies. Using 96-well plate formats, with either individual wells containing unique bacterial species or no-template control wells that had not been spiked with any bacteria, Minich and colleagues were able to demonstrate that well-to-well contamination indeed occurs quite frequently. They found that individual samples were most commonly cross-contaminated by wells in close vicinity, that highly abundant organisms were more likely to be transferred to other wells as contaminants than lower-abundance ones, and that by erroneously introducing additional bacteria to samples, well-to-well contamination impacted measures of diversity. They also found that low-biomass recipient samples were more likely to be affected by this form of contamination than high-biomass ones. Furthermore, they demonstrated that transfer of material from well to well predominantly occurred during the DNA extraction step, with samples that had been processed automatedly using robots showing a greater degree of well-to-well cross-contamination than those that had been processed manually.

These results have particular relevance for decontamination of microbiome data during sequence analysis. One way to account for contamination is to simply remove sequence types that are detected in negative controls, the reasoning being that any sequence type present in the negative control, which should of course have no sequences present as no template DNA was added, must be derived from an external contaminant. However, the work by Minich et al. nicely demonstrates that it is possible for erroneous reads to appear in negative-control samples that are not derived from background kit/reagent contamination. Rather, these are derived from other samples that are present on the same sequencing run, and so simply removing any sequence in a negative control risks removing species that are genuinely present in other samples. Critically, since highly abundant organisms are most likely to be transferred erroneously to other wells, this sort of approach might remove dominant and potentially important members of a microbial community.

Their findings also have relevance for those who wish to use microbiome data for diagnostic purposes. There is significant interest in using sequence profiling to identify microbial biomarkers for a broad range of different environmental and health concerns (14). The work of Minich and colleagues demonstrates that additional care may need to be taken to ensure that biomarkers of interest that are detected in a given sample are truly present and not just derived from well-to-well contamination.

The study also highlights potential concerns with automation. As the costs of sequencing have fallen, microbiome studies have steadily increased in size, with some now incorporating many thousands of samples (2, 15). Clearly, the laboriousness and expense of carrying out all the required processing steps manually make automating parts of this process appealing. However, as the authors suggest, their results indicate that for some critical samples, particularly those that contain low biomass, it may be prudent to consider processing these manually in order to reduce well-to-well contamination.

Finally, although the authors provide many useful suggestions on how to mitigate the impact of well-to-well contamination, their results serve as an additional reminder that, where possible, there is still value in using non-sequence-based approaches to study microbial communities (16). As others have argued previously, results that have been verified and reproduced using multiple different methodologies are more likely to be robust (17).

Regardless, greater understanding of the problem of well-to-well contamination is a welcome development, which should help to guide improvements to microbiome sequencing protocols moving forward.
